# The MYBL1/TCFL5 transcription network: two collaborative factors with central role in male meiosis

**DOI:** 10.1042/BST20231007

**Published:** 2023-11-28

**Authors:** Martin Säflund, Deniz M. Özata

**Affiliations:** Department of Molecular Biosciences, The Wenner-Gren Institute, Stockholm University, S-106 91 Stockholm, Sweden

**Keywords:** A-MYB, meiotic gene expression program, pachytene piRNAs, spermatogenesis, TCFL5, transcription factors

## Abstract

Male gametogenesis, spermatogenesis, is a stepwise developmental process to generate mature sperm. The most intricate process of spermatogenesis is meiosis during which two successive cell divisions ensue with dramatic cellular and molecular changes to produce haploid cells. After entry into meiosis, several forms of regulatory events control the orderly progression of meiosis and the timely entry into post-meiotic sperm differentiation. Among other mechanisms, changes to gene expression are controlled by key transcription factors. In this review, we will discuss the gene regulatory mechanisms underlying meiotic entry, meiotic progression, and post-meiotic differentiation with a particular emphasis on the MYBL1/TCFL5 regulatory architecture and how this architecture involves in various forms of transcription network motifs to regulate gene expression.

## Introduction

**Figure 1.**
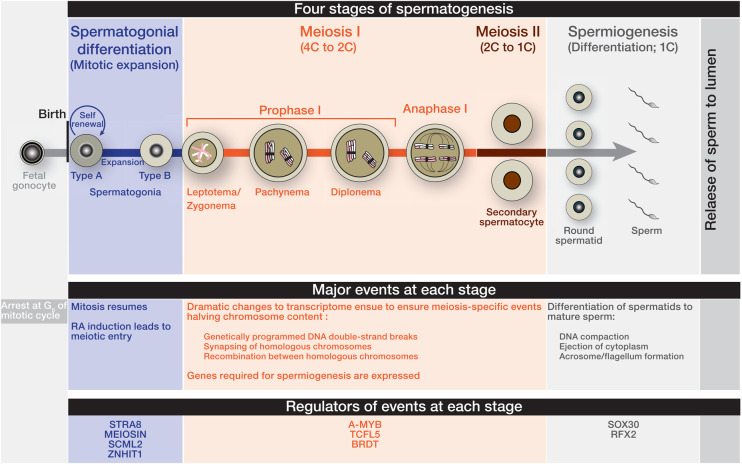
Progression of mouse spermatogenesis that is controlled by key regulators. Fetal male germ cells remain quiescent at G_0_ of mitotic cycle until birth. Thereafter, spermatogenesis resumes with undifferentiated type A spermatogonium cells that have the capacity to self-renew. Later, type A spermatogonia differentiate and expand via mitosis to become differentiating type B spermatogonia. Coinciding with puberty, mitotically dividing type B spermatogonia enter meiosis I in response to retinoic acid (RA). Prophase I is the longest step of meiosis I during which cells undergo complex events, such as programmed DNA double-strand breaks, synapsing and recombination of homologous chromosomes. During anaphase I of meiosis I, homologous chromosomes separate, and cells undergo second meiotic division that ultimately generates haploid round spermatids. Following meiosis, haploid round spermatids differentiate into mature sperms. The prolonged prophase I is accompanied by drastic transcriptome changes controlled by key regulators. Such regulators are expressed spatiotemporally and control the timely expression of genes during entry into meiosis, meiotic progression and spermiogenesis.

Mammalian spermatogenesis is an exquisitely timed process of sperm development comprising of four stepwise stages: (i) self-renewal of spermatogenic stem cells and expansion of spermatogonia via mitosis; (ii) meiotic cell division; (iii) differentiation of spermatids (spermiogenesis) generating mature sperm; and (iv) release of sperm to the lumen of seminiferous tubules [1–3] (Figure 1). In this review, we focus primarily on mice, because mouse testis provides the most abundant and versatile resources for studying mammalian spermatogenesis at genetic and cellular level.

Spermatogenesis starts with the undifferentiated type A spermatogonium cells that retain self-renewing spermatogenic stem cells [4]. The undifferentiated type A spermatogonia later undergo differentiation, during which they lose self-renewal capacity [5] and they rather start expanding via mitotic division [6]. This process gives rise to type B spermatogonium cells that commit to enter meiosis [1–3].

Among the four stages of spermatogenesis, meiosis is, beyond question, the most intricate process that involves series of dynamic events [2]. Upon environmental stimuli, the mitotic program ends and meiotic entry starts [2]: after the pre-meiotic DNA is replicated, male germ cells commit to meiosis with two sequential meiotic divisions that result in haploid gametes (Figure 1). To achieve reductional cell division, several meiotic processes ensue: (i) initiation of genetically programmed DNA double-strand breaks (DBSs); (ii) pairing and synapsing of homologous chromosomes mediated by meiosis-specific scaffold, the synaptonemal complex; (iii) recombination between homologous chromosomes (reviewed in ref. [2]).

Entry into meiosis, progression of meiosis, and meiotic nuclear division and meiotic exit accompany profound transcriptional changes. That is, genes required for such sequential meiotic events are activated spatiotemporally: such as, genes essential for programmed DBSs, synapsing, recombination are expressed at early meiosis, while genes required for meiotic exit are activated later during meiosis. Importantly, meiotic gene expression program is also responsible for the timely activation of genes involved in spermiogenesis [7,8]. Tightly and orderly regulation of meiotic gene expression program is ensured by the collaborative actions of several key transcription factors.

Bolcun-Filas et al. [9] identified the transcription factor, MYBL1 (also known as A-MYB), as a ‘master regulator' of male meiosis using mutagenesis screening: at the start of the meiosis, MYBL1 — highly expressed in testis — promotes the transcription of hundreds of genes essential for the orderly progression of meiosis [9]. MYBL1 contains highly conserved helix-turn-helix DNA binding domain that specifically recognizes DNA consensus sequence, [5′-YAAC(GT)G-3′] [10] We recently revealed that a testis-specific transcription factor TCFL5, which retains basic helix–loop–helix domain and recognizes DNA consensus sequence, [5′-WANSWCGW-3′] [11,12], is also essential for meiosis: MYBL1 and TCFL5 reciprocally reinforce each other's transcription and TCFL5 collaborates with MYBL1 to coordinate meiosis via establishing various distinct transcription network motifs [11,13].

The review discusses how MYBL1/TCFL5 regulatory architecture is regulated and how such architecture then takes part in various forms of transcription networks to timely regulate the meiotic gene expression program in mammals.

## Meiotic entry: from environmental cues to gene expression

Although the mechanical elements of meiosis are widely conserved in females and males, the initiation of meiosis is dimorphic (reviewed in ref. [14]). At ∼13.5 days post coitum (dpc), mitotically dividing female germ cells (oogonia) in developing mouse ovary enter meiosis which is followed by prolonged arrest until just before ovulation. In contrast, embryonic male germ cells stay quiescent at G_0_ of mitotic cycle until birth [14] and at the onset of puberty, mitotically dividing male germ cells (spermatogonia) enter meiosis [14,15] (Figure 1). Even though the timing of meiotic entry is different in both sexes, the environmental stimuli initiating meiosis are partly similar. One common stimulus is the active derivative of vitamin A, Retinoic Acid (RA) plays a curial role in inducing meiotic entry in both sexes [14–16]. Note that specific to females, induction of entry into meiosis also requires BMP signaling in addition to RA [17].

RA diffuses through tissues and binds to retinoic acid receptor (RAR) that heterodimerize with nuclear retinoid X receptor (RXR). RAR–RXR thereafter activates target genes by binding to RA-response elements (reviewed in ref. [14]). Among those RA-responsive genes, the transcription of one key gene encoding Stimulated by Retinoic Acid Gene 8 (STRA8) is activated. Upon activation by RA, the transcription factor STRA8 reprograms the gene expression enabling mitotically active oogonia and spermatogonia enter meiosis [18]. Even though the entry of oogonia into meiosis at 13.5 dpc requires RA stimulation, why is the meiotic entry of spermatogonia delayed until puberty? In embryonic testes, the P450 enzyme, CYP26B1, catabolizes RA into inactive metabolites, thereby preventing spermatogonia from entering meiosis [19]. However, recently, Geyer and his colleagues reported that the initiation of meiosis does not require RA. In RA-deficient mouse testes, the mRNA level of *Stra8* was unaltered in preleptotene spermatocytes and meiotic entry was successfully achieved [20]. Furthermore, in the same study, authors showed that RA is in fact required for type A spermatogonia differentiation during which STRA8 and other genes required for meiotic entry are already expressed [20], suggesting that preparations for meiosis may ensue prior to meiotic initiation.

In addition to the pivotal role of STRA8 in inducing meiotic entry, Ishiguro and his colleagues identified a novel transcription factor MEIOSIN that interacts with STRA8 and orchestrates the meiotic entry by activating the meiotic gene expression program in both sexes [21]. Furthermore, recent study demonstrated that a nucleosome remodeler, zinc finger HIT-type containing 1 (ZNHIT1), is also required for meiotic initiation during spermatogenesis [22]. Although ZNHIT1 is dispensable for *Stra8* transcription, it in fact contributes to the activity of STRA8 by mediating H2A.Z — a histone variant associated with transcription initiation (reviewed in ref. [23]) — incorporation to the promoters of genes occupied by STRA8, thereby helping to initiate their transcription [22]. In the same study, the transcription of *Meiosin* is however affected in the testes of mice lacking ZNHIT1: H2A.Z occupancy around the promoter of *Meiosin* gene was reduced resulting in decreased mRNA level of *Meiosin* in *Znhit1* mutant mice testes [22].

The activation of meiotic gene expression program by RA-responsive STRA8/MEIOSIN axis is central for the mitosis-to-meiosis transition, yet it is equally important to silence mitotic gene expression program. Namekawa and his colleagues identified a germ cell-specific subunit of Polycomb repressive complex 1, SCML2, that suppresses genes related to mitosis by establishing ubiquitination of histone H2A around their proximal promoters [24]. In addition to the epigenetic silencing of proximal promoters of mitotic genes, SCML2 also facilitates the repression of mitotic ‘super enhancers’ (SEs) during mitosis-to-meiosis transition [25]. Note that SEs are aggregates of large number of enhancers whose activities are largely associated with key cell-type-specific genes [26].

## Regulation of meiotic gene expression program

Although we discussed the meiotic entry in both sexes in the preceding section, the focus is on males for the following sections. After entry into meiosis, the first and longest step is prophase I where events of synapsis and recombination take place [2]. Switching from mitosis to prophase I of meiosis induces dramatic transcriptional changes to facilitate such events [8,27,28]. For example, single cell analysis of gene expression during spermatogenesis revealed that mouse spermatocytes of prophase I appear to express significantly larger number of genes compared with other germ cells [8].

Several early transcriptomic studies demonstrated that early prophase I cells — i.e. leptotene (L), zygotene (Z), and early pachytene (ePach) — have almost null transcription [29,30]. This reduced transcription rate is explained by several mechanisms. L, Z, and ePach cells retain paused RNA polymerase II (Pol II) at the proximal promoters of genes that are later engaged into active transcription during mid-late prophase I [31]. Reduced transcription during early meiosis can also be explained by reduced H3K9ac occupancy — which correlates with active transcription in early prophase I cells compared with mid-late prophase I cells [30]. It is recently proposed that dynamic 3D genome reorganization during meiosis impacts the transcription of genes [32]. At the entry into meiosis, topological associated domains (TADs), a key organizational feature of interphase chromosomes, are dissolved. However, meiotic genome starts forming large scale (1–10 Mb) looping interactions [32]. Patel et al. [32] revealed that such interactions are much more pronounced in mid-late prophase I than early prophase I and that actively transcribed loci correspond to those looping loci [32].

Danko et al. [31] and his colleagues recently measured the transcription rate in various stages of prophase I using length-extension chromatin run-on and sequencing (leChRO-seq). They reported that although Pol II physically sits on chromatin, it remained at paused state in early prophase I cells. Yet, upon progression into mid-late prophase I, Pol II is released and became highly active in elongating transcription [31]. This switch from paused Pol II to active elongating Pol II is associated with MYBL1 occupancy that specifically recruits testis-specific bromodomain protein, BRDT, to the promoters of genes [31].

Recently, Maezawa et al. [25] revealed a novel function of MYBL1 in regulating meiotic gene expression program by binding to meiosis-specific SEs thereby ensuring the burst activation of transcription of hundreds of meiotic genes.

Male germ cells lacking MYBL1 fail to develop further than early meiosis underscoring the key regulatory role of MYBL1 [9]. However, MYBL1 binds to the promoters of only one-quarter of genes related to meiosis [9] suggesting that additional transcription factors play a critical role in burst expression of genes during meiosis. We recently identified a testis-specific transcription factor, TCFL5, that collaborates with MYBL1 to promote the transcription of genes required for meiosis, pachytene piRNA and miR-34/449 production, meiotic exit, and spermiogenesis [11,13].

## MYBL1 and TCFL5 establish a positive feedback loop to control each other's transcription

TCFL5 plays a critical role in meiosis and spermiogenesis by activating hundreds of genes related to these pathways [11,13]. The expression of *Tcfl5* is turned on by MYBL1: in mice testes lacking MYBL1, no TCFL5 protein is detected. In the same line of evidence, Cleavage Under Targets and Release Using Nuclease (CUT&RUN) for MYBL1 from FACS-purified male germ cells detected MYBL1 occupancy around the promoter of *Tcfl5* gene [13]. Interestingly, both mRNA and protein levels of MYBL1 are reduced in the testes of *Tcfl5* mutant. Notably, our CUT&RUN for TCFL5 also captured TCFL5 occupancy on *Mybl1* promoter revealing that MYBL1 and TCFL5 reinforce each other's transcription via positive transcriptional feedback [13].

## Transcription network motifs centered around MYBL1/TCFL5 regulatory architecture

**Figure 2.**
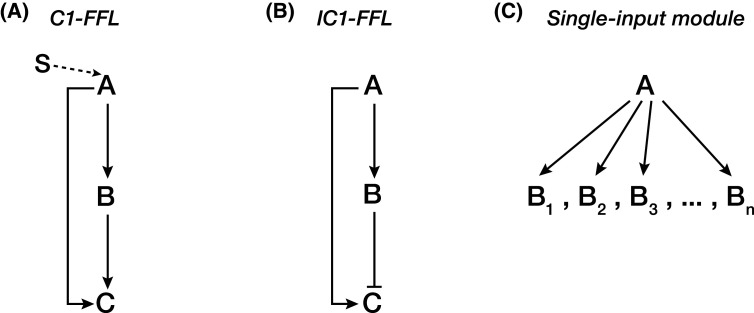
Overview of transcription network motifs in which MYBL1/TCFL5 regulatory architecture takes part. (**A**) *Coherent type 1 feedforward loop (C1-FFL),* upon stimuli, transcription factor A initiates the transcription of downstream transcription factor B. Once the B reaches to its activating threshold, it starts to control its downstream target C whose expression is in parallel amplified by A. (**B**) *Incoherent type 1 feedforward loop (IC1-FFL),* In this motif, A activates the expression of C. Yet in parallel it represses C by activating a repressor B. (**C**) *Single-input module*, this motif is characterized by an activity of a transcription factor that simultaneously controls the activity of large number of genes that function in same biological pathway.

In this section, we will discuss three forms of transcription network motifs established by MYBL1/TCFL5 regulatory architecture that ensures timely expression of meiotic genetic program. However, we will first begin by understanding the general dynamics of these network motifs: (i) coherent type 1 feedforward loop; (ii) incoherent type 1 feedforward loop; (iii) single-input module (Figure 2).

### Coherent type 1 feedforward loop (C1-FFL)

One of the best-studied transcriptional networks is C1-FFL defined by its peculiar genetic architecture: C1-FFL is often initiated by a short signal induction that startlingly results in a delayed stimulation of downstream target gene [33]. That is, once the signal S appears, the activity of transcription factor A, responsive to S, is stimulated. Thereafter, A rapidly binds to the promoter of its downstream transcription factor B. However, for B to activate its downstream gene C, its intracellular concentration must reach the activation threshold. This, therefore, causes a delay in the expression of C, i.e. *sign-sensitive delay* [33]. Once the concentration of B reaches to its activating threshold, it becomes independent of S; even though S is removed and A is quickly inactivated, C is still produced. How is then the activation threshold for B maintained? It is often observed that C in turn binds to the promoter of B to mutually reinforce the expression of B, thereby positive feedback loop may become part of C1-FFL (Figure 2A).

### Incoherent type 1 feedforward loop (IC1-FFL)

In this motif, the transcriptional regulator A activates the expression of C; in parallel, A also represses C by activating the expression of a repressor B (Figure 2B). However, the repressive activity of B is delayed until after the steady-state level of C reaches to a certain threshold. That results in the strong production of C before B represses it, i.e. *response accelerator* [33].

### Single-input module

In this network, a single transcription factor A activates the transcription of a group of target genes [33]. Once A reaches to its activation threshold, it enables the coordinated expression of genes functioning in a certain biological pathway [33] (Figure 2C).

## STRA8/MEISON axis regulates the formation of positive feedback loop between MYBL1 and TCFL5 via C1-FFL

In mice, both mRNA and protein of *Mybl1* precedes that of *Tcfl5* [13]; the time from the appearance of MYBL1 till the expression of TCFL5 is ∼5 days [7]. Although MYBL1 binds to the promoter of *Tcfl5*, why is then TCFL5 expression delayed?

The likely explanation for the delayed expression of *Tcfl5* stems from the dynamical function of C1-FFL established by the STRA8/MEIOSIN axis whose expression is stimulated by RA [18,21]. Here, we will consider RA as an initial cue which appears shortly during entry into meiosis I and initiates transcription factor cascades resulting in the accumulation of TCFL5.

**Figure 3.**
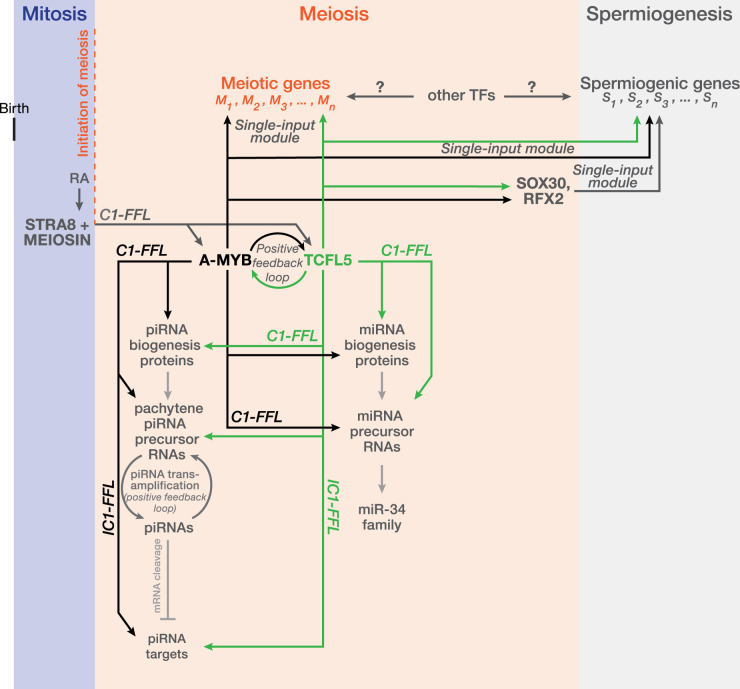
MYBL1/TCFL5 regulatory architecture interacts with various transcription network motifs to regulate meiotic gene expression program. The model highlights the regulatory role of MYBL1/TCFL5 axis established by positive feedback loop in gene expression during spermatogenesis. This model proposes that the expressions of MYBL1 and TCFL5 is regulated by STRA8/MEIOSIN axis via the coherent type 1 feedforward loop (C1-FFL). Once the MYBL1/TCFL5 regulatory architecture is established, it regulates the production of pachytene piRNAs and miRNAs from miR-34 family via C1-FFL, as well as genes required for meiosis and spermiogenesis via a single-input module. The model also highlights that while MYBL1/TCFL5 regulatory architecture ensures the production of pachytene piRNAs, it regulates the transcription of pachytene piRNA targets, organizing an incoherent type 1 feedforward loop (IC1-FFL).

Based on the *sign-sensitive delay* kinetics of C1-FFL model, the STRA8/MEIOSIN rapidly binds to the promoter of *Mybl1* in response to the short RA induction during the entry into meiosis [11,18,21] (Figure 3). However, TCFL5 production is delayed until mid- and late-pachynema stage of meiosis [11], because the intracellular MYBL1 concentration first reaches its activation threshold to drive the transcription of *Tcfl5* [11]. Of note, even though the RA level drops dramatically before pachynema stage of meiosis, TCFL5 is still produced which is because MYBL1 expression is already reached to its activating threshold. We propose that the activating threshold of MYBL1 is in turn maintained by the activity of TCFL5 itself via the positive feedback loop (Figure 3).

## MYBL1/TCFL5 regulatory architecture generates a C1-FFL to ensure massive small silencing RNA production during meiosis

One of the key regulatory elements of meiotic progression and spermiogenesis ensues at post-transcriptional level govern by small silencing RNAs (reviewed in ref. [34,35]). Small silencing RNAs range from 21–30 nucleotides and guide Argonaute family proteins to silence complementary transcripts, i.e. coding and non-coding RNAs [35]. After entry into meiosis I, MYBL1/TCFL5 regulatory architecture activates the expression of two classes of small silencing RNAs: pachytene piRNAs and miRNAs [11,13]. Both pachytene piRNAs and miRNAs play essential role in sperm development (reviewed in ref. [36]). In this subsection, we will discuss how MYBL1/TCFL5 regulatory architecture ensures the abundant production of pachytene piRNAs and miRNAs via C1-FFL.

### Pachytene piRNA production

In the adult testis of placental mammals, male germ cells produce pachytene piRNAs of ∼26 and ∼30 nucleotides in length that guide PIWI proteins to cleave target transcripts [35]. Pachytene piRNAs have immense sequence diversity with >1 million distinct sequences [37,38] and their sequences are not conserved across placental mammals [38,39]. Counterintuitively, they are required for sperm development, even though they evolve rapidly [40,41].

Pachytene piRNAs begin to accumulate at the pachynema stage of meiosis I and become the most abundant group of small RNAs competing for the abundance of ribosomal RNAs [37,38,42]. Pachytene piRNAs are processed from long single-stranded non-coding transcripts, namely pachytene piRNA precursors [37,38,43]. At the onset of pachynema stage of meiosis I, MYBL1 drives the transcription of pachytene piRNA precursors from the well annotated 100 specialized meiotic loci that retain unique genic and epigenetic marks [37,38,44]. In addition to controlling the expression of pachytene piRNAs themselves, MYBL1 drives the transcription of genes encoding piRNA biogenesis factors, such as MILI (PIWIL2) and MIWI (PIWIL1) [37,38]. Together, MYBL1 powers the production of pachytene piRNAs via establishing a C1-FFL (Figure 3).

Importantly, similar to the delayed expression of TCFL5 during early meiosis [11], a delay in the accumulation of pachytene piRNAs from early to middle meiosis is also reported, exemplifying the *sign-sensitive delay* kinetics observed in C1-FFL [37]. Such kinetics presumably prevents background expression of pachytene piRNAs in early meiosis during which intracellular concentration of MYBL1 is likely below the activating threshold.

We recently reported that TCFL5 promotes the transcription of half of the pachytene piRNA genes, as well as genes encoding piRNA biogenesis, thereby establishing C1-FFL [13] (Figure 3). Why is then TCFL5 required for the burst of production of pachytene piRNAs in addition to MYBL1? Among the 100 mouse pachytene piRNA-producing loci, 21 are identified in the genomes of placental mammals [13,44]. That is, those 21 loci produce piRNAs from an evolutionarily conserved location in other placental mammals, namely the old loci. Sun et al. [45] recently proposed a model that pachytene piRNA genes are enriched with structural variations (SVs) and evolution of pachytene piRNA genes converge with that of SVs suggesting that SVs are possible driver of pachytene piRNA gene evolution. In fact, MYBL1 promotes the transcription of old loci, whereas TCFL5 regulates genes that produce pachytene piRNAs only in murine (young loci) [13].

The current model for piRNA biogenesis posits that a piRNA from a particular locus, namely the initiator piRNA, directs the cleavage of piRNA precursor transcripts from other loci or from the same locus resulting in 5′ monophosphorylated pre-pre-piRNAs that are tunneled into further piRNA processing [35]. Thus, piRNAs themselves play pivotal role in the initiation of their own production [35,42,46–48]. Intriguingly, pachytene piRNAs from young loci transcribed by TCFL5 facilitate piRNA production from other loci generally controlled by MYBL1. Similarly, piRNAs from older loci initiate the processing of piRNAs from young loci, thereby ensuring their own production via a positive feedback loop [13]. Such feedback loop controlled by MYBL1/TCFL5 regulatory architecture appears to reinforce massive pachytene piRNA production (Figure 3).

### miR-34 family production

MYBL1/TCFL5 regulatory architecture amplifies the production of miRNAs that belong to miR-34 family (i.e. miR-34b/c and miR-449a/b/c). miR-34 is essential for sperm development [11,49]. While driving the transcription of miR-34 family, MYBL1/TCFL5 regulatory architecture also controls the transcription of six crucial miRNA biogenesis genes: *Drosha*, *Dgcr8*, *Dicer*, *Tarbp2*, *Xpo5*, and *Ago2* [11,49]. Although MYBL1 is expressed in early spermatocytes [13], the accumulation in the abundance of miR-34 family miRNAs is delayed until after this stage [49]. This exemplifies *sign-sensitive delay* kinetics of C1-FFL. That is, even when MYBL1 is turned on, the miR-34 family is slow to turn on suggesting that steady-state abundance of miRNA biogenesis factors must reach a certain threshold to process miRNA precursors into mature miRNAs (Figure 3).

## MYBL1/TCFL5 regulatory architecture and pachytene piRNAs form a IC1-FFL to regulate pachytene piRNA target genes

Mice lacking two of the 100 pachytene piRNA-producing loci, namely *pi6* and *pi18* — producing hundreds-of-thousands of pachytene piRNAs — are infertile [40,41]. PIWI protein guided by a piRNA makes a single scissor-like cut on target transcript that is ultimately destroyed (reviewed in ref. [35]). Using molecular data from mutant and wild-type mice, piRNAs from *pi6* and *pi18* loci direct the cleavage of mRNAs required for sperm function [40,41]. Although those well-defined direct piRNA targets are expressed during meiosis I, the expected increase in their abundance in mutants is not observed during meiosis I, but later during spermatogenesis [40,41]. Pachytene piRNAs are abundantly expressed in meiosis I [37,38,42]. Why is then piRNA-driven mRNA silencing incomplete in meiosis I?

Intriguingly, we recently revealed that in addition to pachytene piRNA production, the transcription of mRNAs, which are to be silenced by pachytene piRNAs from *pi6* and *pi18* loci, are controlled by MYBL1/TCFL5 regulatory architecture [13]. Such a regulatory motif seems to retain a signature of ‘*response accelerator’* IC1-FFL [33]. To this end, the steady-state mRNA abundance of a pachytene piRNA target, which is controlled by MYBL1/TCFL5 regulatory architecture, must first reaches to a specific threshold, which is later repressed by a pachytene piRNA (Figure 3).

## MYBL1 and TCFL5 act via a set of *single-input module* to regulate spermiogenesis

During spermiogenesis, haploid round spermatids undergo complex differentiation program where DNA is compacted, cytoplasm is ejected, acrosome and flagellum is formed resulting in mature sperm (reviewed in ref. [50]). Several studies revealed that large number of genes whose protein products needed during spermiogenesis are expressed during meiosis [11,51,52]. The simultaneous expression of those spermiogenesis-related genes is activated by MYBL1 or TCFL5 exemplifying *single-input module* [9,11]. In this network, a single transcription factor controls multiple genes that often function in a shared pathway [33].

Several other key transcription factors are reported to participate in controlling the expression of genes involved in spermiogenesis. The transcription factor SOX30 expressed in late meiotic and haploid round spermatids controls the expression of genes associated with spermatid differentiation and penetration of sperm through egg plasma [53]. Moreover, the transcription factor RFX2 activates the expression of genes required for sperm motility [54]. Notably, MYBL1/TCFL5 regulatory architecture controls the expression of SOX30 and RFX2 [11], thereby establishing a C1-FFL as part of *single-input module* (Figure 3).

## Conclusion

Spermatogenesis is the developmental process that ultimately produces mature sperm transferring the paternal genome to offspring. Four major steps control sperm development: self-renewal of spermatogenic stem cells and expansion of spermatogonia via mitosis, meiotic cell division, final differentiation of spermatids, and release of sperm to the lumen of seminiferous tubules. In virtually all sexually reproducing organisms, meiosis plays pivotal role to ensure the production of haploid sperm by halving the chromosome content. From the meiotic entry to meiotic exit, changes to gene expression are tightly controlled to prevent dysfunctional gene expression that may lead to infertility. The onset of prophase of meiosis I, MYBL1 is expressed and plays central role in regulation of meiotic gene expression. Importantly, MYBL1 is not alone in this journey; it initiates the production of TCFL5 that in turn amplifies the transcription of *Mybl1*, thereby establishing an interlocking positive feedback loop — MYBL1/TCFL5 regulatory architecture [13] (Figure 3). This regulatory architecture interacts with various forms of network motifs to promote the transcription of genes required for spermatogenesis, including the production of pachytene piRNAs and miRNAs (Figure 3).

**Perspective**

- During male meiosis, two key transcription factors, MYBL1 and TCFL5, establish a central regulatory architecture that enrolls in various forms of transcription network motifs to regulate various pathways required for spermatogenesis.
- MYBL1 binds to SEs which often contain TCFL5 binding motif. How MYBL1 and TCFL5 mechanistically regulate enhancers during spermatogenesis remains unknown. Could MYBL1/TCFL5 re-organize the chromatin to mediate long-range enhancer-gene interactions?
- Although MYBL1/TCFL5 regulatory architecture plays central role via various network motifs, this architecture binds the promoters of half of the meiotic genes [9,13,37] suggesting that other factors possibly contribute to the regulation of gene expression during spermatogenesis.

## Abbreviations

C1-FFL: coherent type 1 feedforward loop
DBSs: double-strand breaks
IC1-FFL: incoherent type 1 feedforward loop
RA: retinoic acid
RAR: retinoic acid receptor
RXR: retinoid X receptor
SEs: super enhancers
STRA8: stimulated by retinoic acid gene 8
SVs: structural variations
TADs: topological associated domains
ZNHIT1: zinc finger HIT-type containing 1
